# Self-Associations Influence Task-Performance through Bayesian Inference

**DOI:** 10.3389/fnhum.2013.00490

**Published:** 2013-08-19

**Authors:** Sara L. Bengtsson, Will D. Penny

**Affiliations:** ^1^Department of Clinical Neuroscience, Karolinska Institute, Stockholm, Sweden; ^2^Wellcome Trust Centre for Neuroimaging, University College, London, UK

**Keywords:** priming, self-esteem, rule task, cognitive control, Bayesian, normative model, computational model

## Abstract

The way we think about ourselves impacts greatly on our behavior. This paper describes a behavioral study and a computational model that shed new light on this important area. Participants were primed “clever” and “stupid” using a scrambled sentence task, and we measured the effect on response time and error-rate on a rule-association task. First, we observed a confirmation bias effect in that associations to being “stupid” led to a gradual decrease in performance, whereas associations to being “clever” did not. Second, we observed that the activated self-concepts selectively modified attention toward one’s performance. There was an early to late double dissociation in RTs in that primed “clever” resulted in RT increase following error responses, whereas primed “stupid” resulted in RT increase following correct responses. We propose a computational model of subjects’ behavior based on the logic of the experimental task that involves two processes; memory for rules and the integration of rules with subsequent visual cues. The model incorporates an adaptive decision threshold based on Bayes rule, whereby decision thresholds are increased if integration was inferred to be faulty. Fitting the computational model to experimental data confirmed our hypothesis that priming affects the memory process. This model explains both the confirmation bias and double dissociation effects and demonstrates that Bayesian inferential principles can be used to study the effect of self-concepts on behavior.

## 1 Introduction

High self-esteem is characterized by thinking well of oneself, whether it is a true or distorted appreciation. Low self-esteem denotes a less consistent and more uncertain regard about one’s abilities (Campbell, 1990). Self-esteem is the evaluative dimension of self-concepts (Harter and Baumeister, 1993). Taking a cognitive architectural approach of personality, self-concepts can be viewed as knowledge structures of attributes of oneself formed from experience and organized as any other mental concept (Markus, 1977; Cervone et al., 2004). They are used to guide the processing of self-relevant information (Kelly, 1955; Markus, 1977), and emerging evidence shows that the way we think about ourselves impacts on aspects such as depression (Harter and Baumeister, 1993), obesity (Ternouth et al., 2009), school performance (Spinath et al., 2006), and criminal behavior (Trzesniewski et al., 2006).

Functional neuroimaging studies have consistently highlighted processes of the anterior medial prefrontal cortex (aMPFC) as part of reflecting upon one’s own character (for a review, see Amodio and Frith, 2006). Enhanced activation is seen when participants judge whether or not traits apply to themselves as compared to when they make judgments about others’ character (Kelley et al., 2002; Mitchell et al., 2002) and subsequently when the traits are high as compared to low in self-relevance (Moran et al., 2006). Furthermore, Macrae et al. (2004) showed that high activation in the aMPFC when judging self-relevant traits resulted in better recollection when debriefed after the experiment of which adjectives had been presented. Bengtsson et al. (2009) found that this area is sensitive to task instructions that make participants specifically monitor their own performance. When told that the task they took was a measure of their ability there was enhanced neural activation in aMPFC when the participants made errors, compared to a group who were told that the task they took was a piloting task. Task difficulty was titrated so that accuracy was matched between the two groups.

Stored in long-term memory, the concept of self may not change extensively over an individual’s lifespan (Marcus and Kunda, 1986; Campbell, 1990). However, the influence of self-concepts on behavior will vary depending on applicability and accessibility of the knowledge structures to the task the individual is encountering (Higgins, 1990). This is exemplified in priming studies where, e.g., priming for “old” makes people more likely to walk slower down the corridor than they would otherwise do (Bargh et al., 1996), or when primed with associations to “professor” people become more likely to score highly on a quiz (Dijksterhuis and van Knippenberg, 1998). Priming refers to the passive and unobtrusive activation of relevant mental representations by environmental stimuli such that people are not and do not become aware of the influence exerted by those stimuli (Bargh and Chartrand, 2000). Dual-process models (Smith and DeCoster, 2000; Strack and Deutsch, 2004) stipulate that human behavior is the result of interactions between automatic/impulsive processes on the one hand and controlled/reflective processes on the other. According to the Strack and Deutsch (2004) model, the Impulsive system is a network of associative nodes, with connections differing in their weight according to how frequently they occur together. Incoming information is always processed by the impulsive system, where the influence of the system on behavior is greatly determined by the extent of pre-activation of specific connections in the associative network. The Reflective system, which focuses attention toward relevant stimuli, is subjected to the individual’s awareness and control. Goal oriented conflict between the two systems costs energy, and can impact cognitive performance, such as results on an IQ-test when conflicts arise between the implicit and explicit self-concept of intelligence (Dislich et al., 2012).

We have previously found that errors on a subsequent working memory task take on a different meaning when participants are primed with associations to “clever” and “stupid.” “Clever”-priming led to increased activity in aMPFC as well as post-error slowing in reaction times, whereas “stupid”-priming was followed by increased activation in insula when the participants made errors and absence of post-error slowing (Bengtsson et al., 2011). Rabbitt (1966) suggested that the slowing of responses immediately after errors is due to the validation of an error, and thus transient changes in response strategy to minimize the possibility of further errors. This proposal is supported by empirical findings that post-error slowing lowers the probability of committing a subsequent error in the post-error trial (Rabbitt, 1966; Danielmeier et al., 2011). Thus, our results suggest that “stupid”-associations led to greater uncertainty as to whether errors had occurred or not. We further speculated that the results may reflect a conflict between the implicit self-associations (e.g., “clever”) and the explicit self-associations (I’m making an error), and that in the case of “clever” the post-error slowing reflects a greater surprise about the outcome. This interpretation is supported by the model of Notebaert et al. (2009), where they propose that post-error slowing represents an attention-grasping (surprising) event. They showed that slowing occurs when the outcome is rare, rather than to errors in particular. When correct responses outnumbered error responses, post-error slowing occurred, whereas when the majority of the trials were incorrect post-correct slowing was observed. In fact, influential theoretical models of self-regulation propose that individuals adjust their behavior so as to minimize the discrepancy between active self-associations and goals. Carver and Scheier (1998) in their model have taken inspiration from control theory and propose that the active self-concept functions as a reference, and in a discrepancy-reducing feedback loop individuals aim to adjust behavior so as to minimize the discrepancy between action and the reference. Similarly, Conway and Pleydell-Pearce (2000) propose that the active self-concept functions as a working memory control process to filter what self-relevant information to encode in order to reduce the tension between active self-knowledge and goals.

The aim of the present study is to test our predictions from Bengtsson et al. (2011): priming with associations to “clever” leads participants to treat errors as more surprising than when they are primed “stupid,” since an error would then generate a larger discrepancy between expectations and outcome. If this is true, using the same logic, we would also expect a greater surprise to correct responses when participants are primed with “stupid”-associations. We examined these predictions in a behavioral study using a rule-association task (Crone et al., 2006). Additionally, we develop a computational model to improve the understanding of underlying mechanisms. We use Bayesian probability theory to test if behavior may be regulated based on the probabilistic attribution of outcomes to a subject’s own abilities. The aim of the model is to shed light on what we mean by various concepts such as self and esteem. Previously, Bayesian theory has proven useful in understanding brain and behavior on many levels (Doya et al., 2007), from sensory perception (Ernst and Banks, 2002) to motor learning (Kording and Wolpert, 2004) and social interaction (Yoshida et al., 2008) (for a recent review, see Penny, 2012). However, to our knowledge, there has to date been no research on using Bayesian inference to probe the mechanisms relating trait-associations to behavior.

Our model makes the following assumptions (a) that our behavioral task embodies two processes (i) memory: remembering a rule for how to behave and (ii) response accumulation: integrating stimuli with rule memory to produce an appropriate response, (b) the decision threshold for the response accumulation process, β, adapts over trials by switching to a higher value if accumulation was inferred to be incorrect on the previous trial, (c) mean reaction time is proportional to a log-odds ratio (log β/[1 − β]), (d) estimates of memory integrity (the probability of correctly remembering the rule) are updated over time. Additionally, we hypothesize that priming affects the memory process and test this hypothesis by fitting our model to subjects’ behavioral data. This hypothesis stems from the notion that both working memory and priming are considered to be top-down processes where they both depend on goal-directed processes that rely on previous knowledge. The response accumulation process can be considered a bottom-up process since it relies on sensory stimuli (Pessoa and Ungerleider, 2004).

## 2 Materials and Methods

This section first describes the participants involved in the study and the behavioral task they performed. The “Bayesian Model” section then describes the assumed component processes underlying the task, and how the probability of them failing relates to incorrect task performance. It also describes how the decision threshold for the accumulation process is adaptively switched between low and high levels, and how reaction time is related to this threshold. The section on “Model Likelihood” describes how the experimental data (time series of error/correct outcomes and reaction times) are related to model parameters such as memory integrity and decision thresholds. Finally, the “Model Fitting” section describes how the model is fitted to the experimental data.

### 2.1 Participants

Fifteen native English speaking volunteers (aged 24.7 ± 4.1 years; 8 females) took part in the study. In addition to these participants three subjects were tested but excluded because of technical failure or performance below chance-level. The participants all gave written informed consent, and the study was approved by the joint ethics committee of the Institute of Neurology and University College London Hospital, London, UK.

### 2.2 Stimuli and task description

Each subject took part in a total of six experimental sessions, and each session comprised a priming part followed by a rule-association part. We used a within-subject design where each participant was primed both with associations to “clever” and “stupid.” The order of the priming categories was counterbalanced between participants; the participants begun with either three consecutive sessions that involved the “clever” prime or three consecutive sessions that involved the “stupid” prime. The participants were primed using the scrambled sentence task (Bargh and Chartrand, 2000). In our study, each scrambled sentence consisted of six words and participants judged whether or not it could be made into a grammatically coherent sentence by using five of the six words. The participants responded “yes” by pressing a button corresponding to their right index finger or “no” by pressing a button corresponding to their right middle finger on a button-box. Each sentence was presented for 8000 ms during which time the participant had to respond. In each session, 70% of the sentences had words that were synonyms for either “clever” or “stupid,” and 30% of the sentences were neutral. The neutral sentences were introduced in accordance with the description of Bargh and Chartrand (2000), with the aim to disguise the purpose of the language task. Examples of sentences in the “clever” condition are “pupil intelligent Todd and his pencil” and “the brightest nothing idea everything promoted,” and examples of sentences in the “stupid” condition are “welcome not morons one are here” and “the room obtuse had white green.”

**Figure 1:**
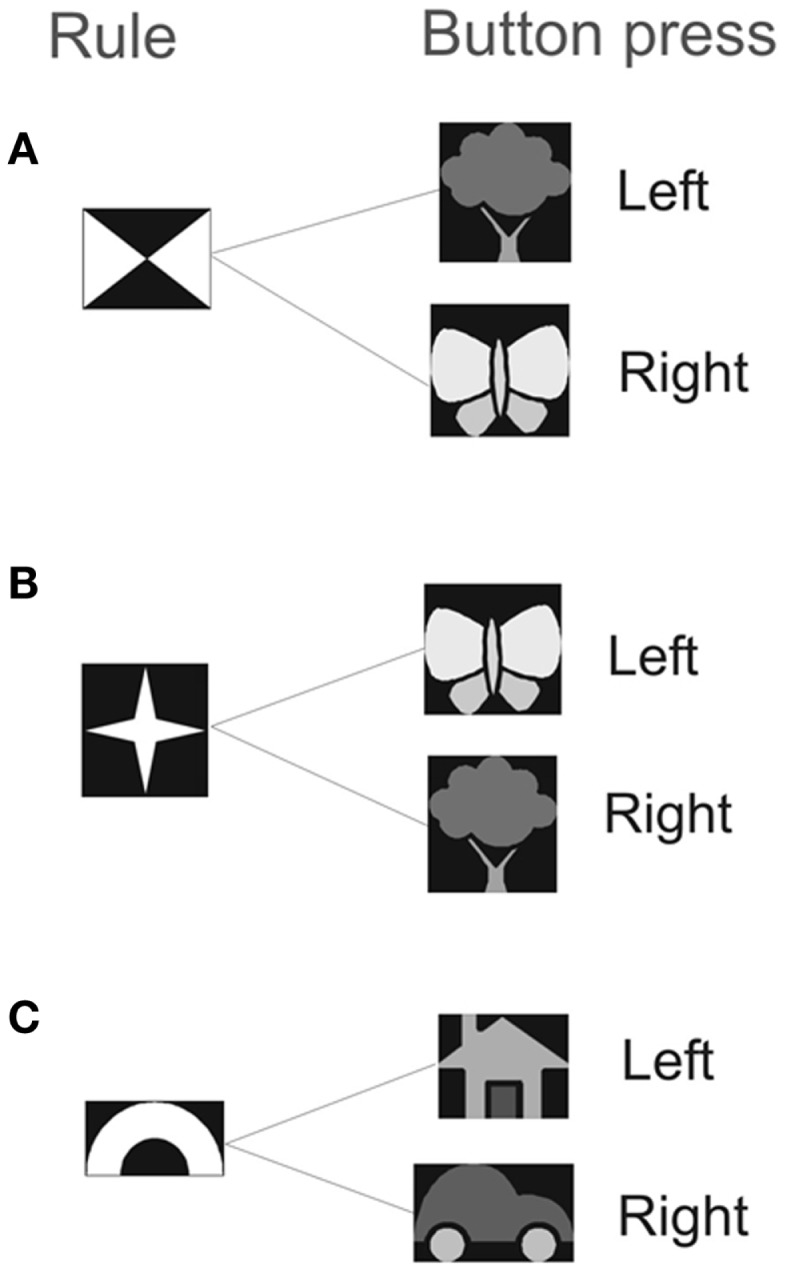
**The experiment consisted of two rule types: (A) and (B) indicate the bivalent rule, (C) indicates the univalent rule**. Participants viewed the rule cue for 1 s. After a 0.5 s delay the target stimulus was presented for 2.5 s. The response was either a left or a right button press, depending on the relevant mapping that had been previously learnt.

We measured the effect of the priming on response time (RT) and error-rate on a computer-based rule-association task (Crone et al., 2006) (Figure 1). In this task, participants were asked to respond to targets that could be either “bivalent” or “univalent.” Bivalent targets refer to visual targets that were associated with different responses depending on which of two rules is currently relevant. The outcome of the response target was to either press the left or the right button. The univalent target was associated with fixed responses. A rule cue was presented on a computer screen for 1000 ms, this was followed by a blank screen for 500 ms before the response cue appeared. A pause of 2000–8000 ms occurred before the next rule cue was presented. For example, if the rule cue consisting of four triangles (Figure 1) was followed by a butterfly (response cue) the participant should press the right button on the keypad, whereas if the star appeared as the rule cue (Figure 1) and was followed by a butterfly (response cue) the participant should press the left button. The task consisted of a distribution of 70% bivalent cues and 30% univalent cues in randomized order, which gives roughly the same number of presentations of each rule cue. There was no particular hypothesis for modulation of expectancy. We followed approximately the distribution used in Crone et al. (2006).

Eight scrambled sentences were presented followed by a sequence of 50 rule trials. This constitutes a session and there were three consecutive sessions for each prime (clever and stupid). Prior to data collection, participants practiced the rule task for 80 trials, and the language task for 20 trials, with all the sentences being of neutral character. The data was analyzed using custom written Matlab scripts (Matlab r2010a, The Math Works, Natick, MA, USA). The participants performed the task inside an fMRI-scanner and performed a second task after the above described paradigm but these data will be presented elsewhere.

To disguise a link between the two tasks, we told the participants that we would alternate between a language task and a rule task. Our explanation was that the experimenters had long experience of participants getting bored during experiments, and this was a way to prevent this from happening. After the experiment the participants were debriefed as to whether they thought any of the tasks would influence performance on the other task, and whether they noticed any theme in the sentences. The debriefing was adapted from Bargh and Chartrand (2000) to fit with the present tasks. None of the participants reported any link between the two tasks. One participant reported that the sentences had either a positive or a negative character, another participant reported that some sentences had words related to “clever” in them. However, none of these participants reported any understanding that one task would influence the other. Therefore, these participants were included in the analysis. After the experiment the participants also filled out the Rosenberg Self-esteem scale (Rosenberg, 1965), which is a 10-item questionnaire measuring the participant’s general explicit self-esteem, and the State-Trait Anxiety Inventory (STAI; Spielberger et al., 1983) which consists of 40 questions on anxiety.

### 2.3 Behavioral data analysis

As mentioned in the Section “Introduction,” a delay in RT on a correct trial after an error is often observed in cognitive task performance, and is suggested to reflect participants’ control over behavior (Rabbitt, 1966). In the present paper we consider two types of trials; correct following correct (CC) and correct following error (EC). Reaction times on CC trials are referred to as RTs after correct, and on EC trials as RTs after error. We do not consider RTs on error trials themselves because generally these may vary substantially, without known cause.

First we looked at the overall RTs for CC trials and EC trials respectively. We then organized data from “early” and “late” trials using an epoch length of *N*_CC_ for CC trials and an epoch length of *N*_EC_ for EC trials. Here “epoch length” refers to the number of trials that define the early and late periods. Fewer trials are used for the EC category due to the smaller number of errors than corrects. We present results obtained with *N*_CC_ = 20 and *N*_EC_ = 3 although our effects are robust over a range of parameters. This means that the first twenty CC trials of the first session were compared with the last twenty CC trials of the third session for each priming category (clever/stupid), and the first three EC trials of the first session were compared to the last three EC trials of the third session for each priming category. The data was compared using Student’s paired *t*-tests.

We investigated accuracy for each priming category by computing the mean error rates in percent for each of the three sessions, and made pairwise comparisons between sessions within a priming category as well as between priming categories using two-tailed paired *t*-tests.

We also investigated whether there was any correlation between RT and correct rate. RT was first aggregated over participants with subject means subtracted. We then regressed these RTs onto error rate, using data from sessions 1 and 3.

In addition, we investigated if there was any correlation between scores on the psychometric questionnaires (Rosenberg’s self-esteem questionnaire and the STAI) and the difference in error rate between late and early sessions after “stupid”-priming, the mean error-rate after “clever”-priming, as well as the difference in RT between late and early sessions after “stupid”-priming.

### 2.4 Computational model

#### 2.4.1 Rule-association task

Here we describe a model of the bivalent trials of the rule-association task. We focus on the bivalent, rather than the univalent trials, as the latter were not affected by priming (see Results).

For the bivalent trials participants must remember and act on a rule. For rule A, participants should press the left button when the tree cue appears and the right button when the butterfly cue appears. For rule B, participants should press the right button when the tree cue appears and the left button when the butterfly cue appears. Which rule is active is indicated by one of two rule symbols presented earlier (Figure 1). Success requires that neural circuits in the motor system integrate information from working memory about which rule is active (A or B) with information from the visual system about which cue is present (tree or butterfly). We call these two processes “memory” and “response accumulation.” The term accumulate refers to Evidence Accumulation (EA)-type models established in decision theory in which evidence is accumulated until a threshold is reached and an action is triggered (Gold and Shadlen, 2001). These models are reviewed in Bogacz et al. (2006). We denote successful rule memory with *m_t_* = 1 and successful response accumulation with *c_t_* = 1 where *t* refers to trial number. We denote the probabilities of these events as

(1) $$\begin{array}{l} p(m_{t}=1)=\pi \\ p(c_{t}=1)=\beta_{t} \end{array}$$

We also refer to π as memory integrity. The quantity β*_t_* is also referred to as the decision threshold (see below).

**Figure 2:**
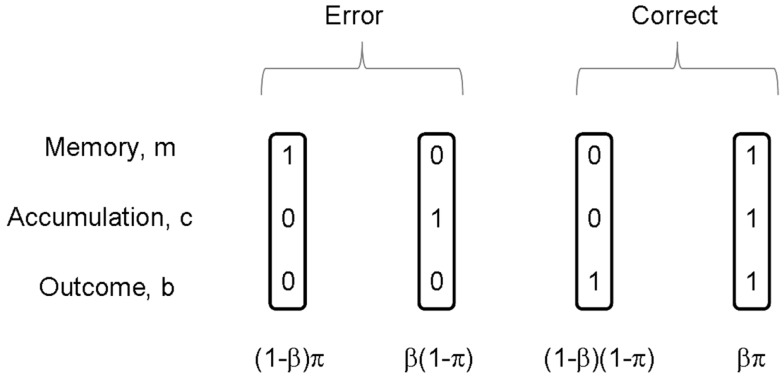
**On each trial, memory *m* of the rule is either correct or incorrect, and accumulation *c* is either correct or incorrect**. The figure shows the four possible combinations of these events. There are thus two ways in which a correct outcome can be produced, and two ways in which an incorrect outcome (error) can be produced. If π is the probability of correct memory, β the probability of correct integration, and these two events are independent, then the probabilities of the joint events *p*(*m*, *c*) are given as in the figure.

Both of the processes being correct leads to a correct outcome on that trial, *b_t_* = 1. Importantly, we note that a correct outcome can also be achieved by incorrect memory *m_t_* = 0 and incorrect cue integration *c_t_* = 0, i.e., a “fluke.” If the two processes are independent then the probability of the various combinations *p*(*m*, *c*) follows from the standard rules of probability theory (Wackerly et al., 1996) as shown in Figure 2. The probability of a correct outcome is given by

(2) $$\begin{array}{ccc} r_{t} & =p\left(b_{t}=1\right) & \\ & =\beta_{t}\pi +\left(1-\beta_{t}\right)\left(1-\pi \right) \end{array}$$

We assume that our normative participant knows the outcome of a trial. In our experiment no explicit feedback was given to the participants as to whether they were correct or incorrect on a given trial. However, on tasks that encourage quick responses participants are often aware of making an error at the time they respond (Rabbitt, 1966). We also know from imaging studies that there is error related activation in the mid-anterior cingulate cortex (mid-ACC) when people make errors without receiving external feedback (Bengtsson et al., 2011). We therefore assume that the normative subject has access to this information.

Our normative subject does not, however, know whether memory for the task or response accumulation were correct on that trial. They can however infer the probabilities of these events using Bayes rule (Bernardo and Smith, 1993). After a given outcome, the probability that response accumulation was incorrect can be computed. These *posterior* probabilities are given by Bayes rule and can be read off from Figure 2.

(3) $$\begin{array}{ll} & p\left(c_{t}=0|b_{t}=0\right)=\frac{\left(1-\beta_{t}\right)\pi }{\left(1-\beta_{t}\right)\pi +\beta_{t}\left(1-\pi \right)}\; \end{array}$$

(4) $$\begin{array}{ll} & p\left(c_{t}=0|b_{t}=1\right)=\frac{\left(1-\beta_{t}\right)\left(1-\pi \right)}{\left(1-\beta_{t}\right)\left(1-\pi \right)+\beta_{t}\pi }\; \end{array}$$

#### 2.4.2 Adaptive decision threshold

A consistent finding in the decision-making literature is that individuals generally slow down their response following an error so as to regain control over behavior (Rabbitt, 1966). In the context of evidence accumulation models of decision making, one mechanism for delaying responses is to increase the decision threshold. Indeed, the idea that decision thresholds are adaptively updated has been explored in the decision-making literature (Bogacz et al., 2006; Simen et al., 2009). These adaptive thresholding processes have been studied in the context of simple two-alternative forced choice (2AFC) tasks (for a review, see Bogacz et al., 2006). One algorithm for adapting the decision threshold is to decrease it if the previous trial was correct and increase it if the previous trial was incorrect (Myung and Busemeyer, 1989). However, such an approach is not straightforwardly implemented in more complex decision tasks.

For example, in the rule-association task used in this paper, an incorrect trial outcome may not be due to incorrect evidence accumulation. An incorrect outcome may rather be due to faulty working memory. Therefore, before deciding whether to increase or decrease the decision threshold it is necessary to *infer* whether the accumulation process was correct or incorrect. We propose that, for normative subjects, this inference is made using Bayes rule and refer to the resulting process as Bayesian Adaptive Thresholding (BAT).

In this paper we use a simple two-state model for this adaption process which provides two levels of decision threshold (or “accumulation success”); low and high, denoted by β[1] and β[0]. If response accumulation was inferred to be incorrect on the current trial then the decision threshold for the next trial should assume the high level. Similarly, if it was inferred to be correct then a low threshold will be used on the following trial. This is the specific BAT process assumed in this paper. The price to pay for using a high threshold is that the response will be delayed and this relation can be quantified using a reaction time model (see next section). One might also conceive of a BAT process in which β*_t_* is continuously updated. We have, however, focused on a discrete model as it relates more directly to the behavioral results (specifically the early to late double dissociation reported in Section 3.1.3).

To incorporate the adaptive threshold into our model we substitute β*_t_* = β[*c_t_*_-1_] into equations (2–4). For example, the outcome probability from equation (2) becomes

(5) $$p\left(b_{t}=1|c_{t-1}\right)=\beta \left[c_{t-1}\right]\pi +\left(1-\beta \left[c_{t-1}\right]\right)\left(1-\pi \right)$$

This shows that the outcome on the current trial depends on whether participants believed response accumulation was successful on the previous trial. In Section 2.5 below we show how this relation can be used to write down the likelihood of an outcome sequence. This quantity is necessary for estimating model parameters from data.

#### 2.4.3 Reaction time model

We use an Evidence Accumulation (EA)-like model to describe the process of integrating working memory with sensory input (Gold and Shadlen, 2001). EA or Drift Diffusion Models (DDMs) describe how evidence is accumulated until a threshold is reached and then an action is triggered. Specifically, the quantity that is accumulated is the log odds ratio, log *p*/[1 − *p*] where *p* is the probability with which it is believed one should make a specific response (e.g., left button press). These models are known to be optimal for 2AFC decision tasks (Bogacz et al., 2006). Gold and Shadlen (2001) review a large body of work in which neural firing rates on 2AFC tasks are seen to correlate with log odds ratios.

For the rule-association task employed in this paper it is not clear, however, that such simple EA models are optimal. Recently Yu et al. (2009) have described a normative model of the Eriksen Flanker task, and in later work (Liu et al., 2009) they also provide a connection to DDMs from which they derive semi-analytic formulae for reaction times and error rates. We note that a similar approach is possible for the bivalent rule-association task where, mathematically, the rule cue rather than the flankers act as the “context” variable. The univalent rule-association task is a 2AFC task. Switching between the univalent and bivalent conditions requires an additional task-switching process.

**Figure 3:**
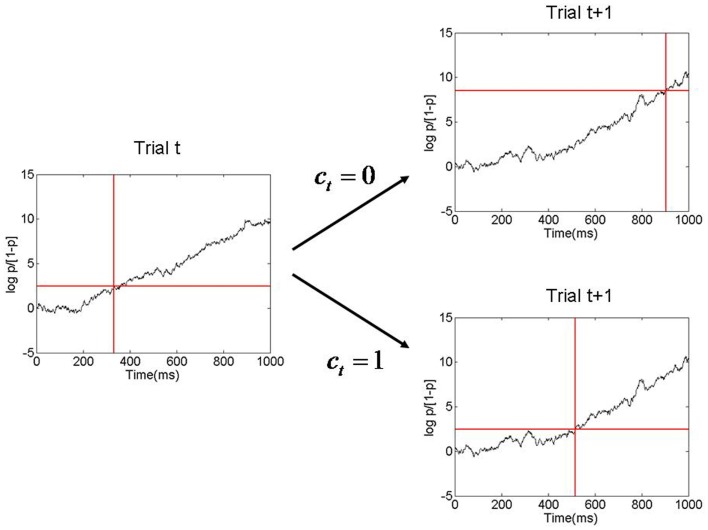
**Figure showing evidence accumulation process and how the decision threshold is changed from one trial to the next**. Here *p* is the probability of pressing the right button and log *p*/[1 − *p*] is the corresponding log odds ratio. In this example the decision threshold has a low value on trial *t*, corresponding to *p* = β[1]. Evidence is accumulated and a response is made when the threshold is reached, at about 350 ms. After the response, an inference is made as to whether response accumulation was correct on that trial (*c_t_* = 1). If it was deemed correct then the threshold remains low on the next trial. Otherwise it is increased (corresponding to *p* = β[0]), resulting in a longer RT on trial *t* + 1. This process is referred to as Bayesian Adaptive Thresholding.

We have outlined how the above approach can be applied to the rule-association task in ongoing, unpublished work (Bengtsson and Penny, 2013). This has motivated us to assume that the average reaction time is proportional to a log-odds ratio threshold (this is the case for simple EA models and the approach described in Bengtsson and Penny, 2013). That is, the likelihood of RT data, *y*, is given by

(6) $$\begin{array}{llll} p\left(y_{t}|c_{t-1}\right) & =N\left(y;\mu_{y},\sigma_{y}\right)\;\\ \mu_{y} & =\mu \theta_{T}\; & & \;\\ \theta_{T} & =\log\left(\frac{\beta \left[c_{t-1}\right]}{1-\beta \left[c_{t-1}\right]}\right)\; & & \; \end{array}$$

where μ is the evidence accumulation slope, and θ*_T_* is the log-odds ratio threshold. Larger β values produce higher θ*_T_*’s and thus longer RTs. The β[0] and β[1] values described earlier therefore produce a short and a long average RT, as depicted in Figure 3.

#### 2.4.4 Me-Focus

A complementary view on the inferential process subsequent to an outcome is the extent of “me-focus.” This is the extent to which a subject identifies themselves with the outcome of a trial. In our experimental context we hypothesize that the self is most strongly associated with the memory process. The extent of “me-focus” can therefore be quantified by the probability *p*(*m_t_* = *b*—*b*). For correct and incorrect outcomes these are given by

(7) $$\begin{array}{ll} & p\left(m_{t}=0|b_{t}=0\right)=\frac{\beta_{t}\left(1-\pi \right)}{\left(1-\beta_{t}\right)\pi +\beta_{t}\left(1-\pi \right)}\; \end{array}$$

(8) $$\begin{array}{ll} & p\left(m_{t}=1|b_{t}=1\right)=\frac{\beta_{t}\pi }{\left(1-\beta_{t}\right)\left(1-\pi \right)+\beta_{t}\pi }\; \end{array}$$

We conclude this section with a brief summary of the model assumptions. We have assumed (a) that our behavioral task embodies two processes (i) memory: remembering a rule for how to behave and (ii) response accumulation: integrating stimuli with rule memory to produce an appropriate response, (b) the decision threshold for the response accumulation process adapts over trials by switching to a higher value if accumulation was inferred to be incorrect on the previous trial, (c) mean reaction time is proportional to a log-odds ratio (log β/[1 − β]). The adaptive thresholding procedure follows from an application of Bayes rule (Bernardo and Smith, 1993) and the reaction time model is based on similar properties of 2AFC (Bogacz et al., 2006) and contextual decision-making tasks (Liu et al., 2009).

### 2.5 Model likelihood

This section describes how the behavioral data on error rates and reaction times can be related to model parameters such as memory integrity and accumulation thresholds.

#### 2.5.1 Outcome likelihood

The outcome on the current trial depends on whether we believed accumulation was correct on the previous trial. This in turns depends on the outcome of that trial and whether we believed accumulation was correct on the trial before that. The probability of an outcome sequence comprising, for example, *T* = 3 trials *b* = {*b*_1_, *b*_2_, *b*_3_}is therefore given by the product

(9) $$p\left(b|\pi ,\beta \right)=p\left(b_{3}|b_{2},b_{1}\right)p\left(b_{2}|b_{1}\right)p\left(b_{1}\right)$$

where

(10) $$\begin{array}{l} p(b_{3}|b_{2},b_{1})=\sum_{c_{2}}\sum_{c_{1}}p(b_{3}|c_{2})p(c_{2}|c_{1},b_{2})p(c_{1}|b_{1})\\ p(b_{2}|b_{1})=\sum_{c_{1}}p(b_{2}|c_{1})p(c_{1}|b_{1}) \end{array}$$

As the sequence grows in length one can see that computation of the likelihood becomes exponentially expensive, because the number of terms in equation (10) grows as 2^(^*^T^*^ − 1)^.

#### 2.5.2 Low-order approximation of outcome likelihood

However, it turns out that these (*T* − 1)th-order conditional probabilities can be adequately approximated by lower-order conditional probabilities. We use the first order approximation

(11) $$p\left(b_{t}|b_{t-1},..,b_{1}\right)\approx p\left(b_{t}|b_{t-1},c_{t-2}=1\right)$$

That is, by assuming that accumulation was correct on the trial before last (its more likely to be correct than not, unless π and β are very low). Under this approximation the outcome on trial *t* then depends on the outcome at *t* − 1 only

(12) $$p\left(b_{t}|b_{t-1},c_{t-2}=1\right)=\sum_{c_{t-1}}p\left(b_{t}|c_{t-1}\right)p\left(c_{t-1}|c_{t-2}=1,b_{t-1}\right)$$

assuming *c*_0_ = 1. The likelihood of a sequence of outcomes, *b*, is then given by

(13) $$p(b|\pi ,\beta )\approx p(b_{1})\prod_{t=2}^{T}p(b_{t}|b_{t-1},c_{t-2}=1)$$

#### 2.5.3 Joint likelihood

The likelihood of outcomes, RTs, and integration sequence is given by

(14) $$p\left(b,y,c|,\pi ,\beta \right)=\prod_{t=1}^{T}p\left(b_{t}|c_{t-1}\right)\prod_{t=1}^{T}p\left(c_{t}|c_{t-1},b_{t}\right)\prod_{t=1}^{T}p\left(y_{t}|c_{t-1}\right)$$

From this we can compute the joint likelihood of outcomes and RTs

(15) $$p\left(b,y|,\pi ,\beta \right)=\sum_{i=1}^{2^{T}}p\left(b,y,c_{i}|,\pi ,\beta \right)$$

and the likelihood of an integration sequence

(16) $$p\left(c_{i}|b,y,\pi ,\beta \right)=\frac{p\left(b,y,c_{i}|,\pi ,\beta \right)}{p\left(b,y|,\pi ,\beta \right)}$$

Equation (15) again involves an exponentially expensive summation. But we can use the same low-order approximation as before, this time to approximate the joint likelihood. This lower-order approximation has been validated by comparing exact and approximate likelihoods on short data sequences (e.g., *T* = 10). We have also generated synthetic data and found that the approximate likelihood is maximized by values that are very similar to the true known parameter values.

#### 2.5.4 Reaction time likelihood

We can integrate out the variable *c_t_*_ − 1_ to see how reaction time is dependent on the outcome of the previous trial (assuming that integration was correct on the trial before that)

(17) $$p(y_{t}|b_{t-1},c_{t-2}=1)=\sum_{c_{t-1}}p(y_{t}|c_{t-1})p(c_{t-1}|,c_{t-2}=1,\;b_{t-1})$$

### 2.6 Model fitting

The following model fitting procedure used a Bayesian estimation algorithm (Gelman et al., 1995) to estimate model parameters from behavioral data. The work in this paper is therefore Bayesian in two ways (i) providing a computational model of subject behavior and (ii) estimating the parameters of that model from data.

#### 2.6.1 Fitting group data

We fit the Bayesian model to data from the group of participants as follows. We focus on a main empirical finding of the paper; that for the stupid prime, the RTs after correct responses are negatively correlated with correct response rate (see Section 3.1.2). We used the model to regress RTs *y* onto correct rates, *r:* first, we inverted equation (2) to write π as a function of β and *r*

(18) $$\pi \left(\beta ,r\right)=\min\left(1,\frac{r-1+\beta }{2\beta -1}\right)$$

where the *min* operator is required to ensure that π < 1. The value of β to be used depends on whether or not response accumulation was inferred to be correct on the previous trial. But for the purpose of the group model fitting we used the approximation β = β_1_ (accumulation assumed correct on previous trial, i.e., *c_t_*_ − 1_ = 1). Second, we used equation (17) to relate β[1], β[0], μ, and π(β, *r*) to expected RT (this integrates over *c_t_*_ − 1_ but assumes *c_t_*_ − 2_ = 1). Model fit was then assessed using the squared difference between these expected RTs and the actual RTs. Log model likelihood was defined equal to negative model error. We then employed a Bayesian estimation procedure with uniform priors

(19) $$\begin{array}{llll} p\left(\beta \left[1\right]\right) & =U\left(0.5,1\right)\;\\ p\left(\beta \left[0\right]\right) & =U\left(0.5,1\right)\; & & \;\\ p\left(\mu \right) & =U\left(50,1000\right)\; & & \; \end{array}$$

where *U*(*a*, *b*) denotes a uniform density with minimum and maximum values *a* and *b*. The posterior parameter density *p*(β[1], β[0], μ—*y*, *r*) was then estimated using a Metropolis-Hastings algorithm (Gelman et al., 1995) with 20,000 iterations. The first 10,000 samples were discarded to accommodate burn-in effects. The remaining 10,000 samples then comprise our approximation to the posterior density.

#### 2.6.2 Fitting individual subject data

We fitted the model to both RT and outcome data from the first and last sessions. This was implemented separately for each subject and type of priming (stupid or clever). The memory integrity variable π was allowed to be different for the two sessions; π_1_ (first) and π_2_ (last). The likelihood of a model was approximated as described above. We then employed a Bayesian inference procedure with uniform priors

(20) $$\begin{array}{llll} p\left(\pi_{1}\right) & =U\left(0,1\right)\;\\ p\left(\pi_{2}\right) & =U\left(0,1\right)\; & & \;\\ p\left(\beta \left[1\right]\right) & =U\left(0.9,0.94\right)\; & & \;\\ p\left(\beta \left[0\right]\right) & =U\left(0.999,1\right)\; & & \;\\ p\left(\mu \right) & =U\left(310,420\right)\; & & \; \end{array}$$

where the priors over β[1], β[0], and μ are constrained by the results of the group analysis (see Section 3.2.3). The posterior parameter density was then estimated using the Metropolis-Hastings algorithm with 20,000 iterations. The first 10,000 samples were again discarded to accommodate burn-in effects. We also computed the posterior mean for each subject and type of priming and used two-way paired *t*-tests to test whether memory integrity varied over sessions. This was implemented separately for the stupid and clever prime data. We hypothesize that priming affects memory integrity.

## 3 Results

### 3.1 Behavioral results

We first analyzed the univalent trials, and found no significant difference between the two primes in the number of errors; stupid (6.9 ± 1.8) and clever (6.0 ± 0.6). All the data analyses and modeling that follow therefore relate to the bivalent trials.

#### 3.1.1 Error rates by session

**Figure 4:**
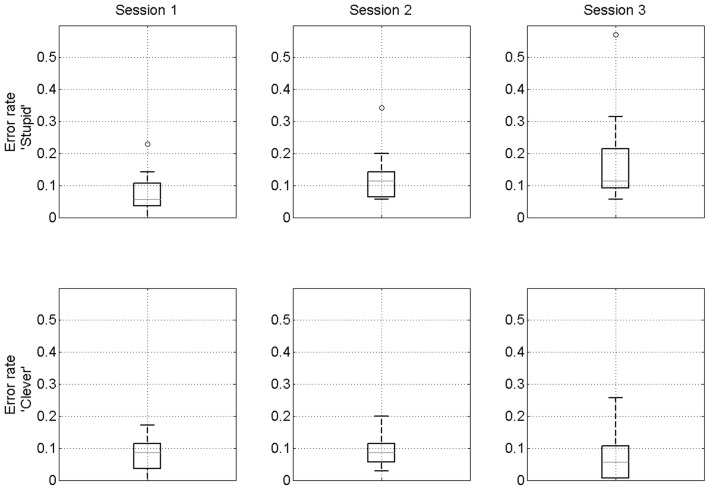
**Confirmation bias**. Boxplots of error rates over participants for “stupid” prime (top row) and “clever” prime (bottom row). On each box, the red line indicates the median, the edges of the box are the 25th and 75th percentiles, the whiskers extend to the most extreme data points considered not to be outliers, and outliers are plotted individually. The outlying data points in the top row are all from participant 10.

When participants are primed “stupid” the mean error rates are 7, 12, and 18% for sessions 1–3. They are significantly different between sessions 1 and 2 (*p* = 0.01, *t* = 2.98, *df* = 14), 2 and 3 (*p* = 0.02, *t* = 2.6, *df* = 14), and 1 and 3 (*p* < 0.01, *t* = 3.37, *df* = 14). Thus, we observed that when “stupid”-associations are evoked participants’ performance becomes increasingly worse whereas when participants are primed “clever” the mean error rates are 8, 9, and 8% for sessions 1–3 (no significant differences). We refer to this as a confirmation bias. Additionally, we find that only in session 3 are stupid error rates significantly higher than clever error rates (*p* < 0.01, *t* = 3.54, *df* = 14). Boxplots of these effects are shown in Figure 4. These effects remain significant if the outlying participant (participant 10 – see small circles in top row of Figure 4) is removed.

#### 3.1.2 Stupid prime: reaction time versus error rate

Since we had observed that the correct rate was deteriorating over time when participants had been primed stupid we investigated whether there was any correlation between RT and correct rate for this condition. RT was first aggregated over participants with subject means subtracted. We then regressed these RTs onto error rate, using data from sessions 1 and 3. For RT after errors we obtained (*r* = −0.03, *p* = 0.81) and for RT after corrects (*r* = −0.41, *p* = 0.001). For every percentage point decrease in correct rate there is a 5 ms increase in RT (after corrects). The same pattern of results was found using data from all sessions and after removing an outlying participant.

#### 3.1.3 Reaction times in early versus late epochs

**Figure 5:**
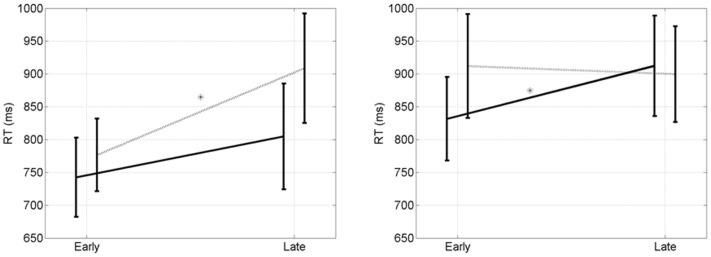
**Early to late double disocciation**. (Left) RT (ms) after errors. There is a significant early to late increase when participants are primed “clever” (*p* = 0.01, dotted line, *), but not when they are primed “stupid” (*p* = 0.22, solid line). (Right) Reaction times (ms) after corrects. There is a significant early to late increase when participants are primed “stupid” (*p* = 0.03, solid line, *), but not when they are primed “clever” (*p* = 0.39, dotted line). Error bars indicate the standard error of the mean.

We now focus on the early and late epochs for both priming conditions. The overall mean RT is 848 ms. RTs for clever are significantly longer than for stupid (874 ms versus 822 ms, *p* = 0.03, *t* = 2.03, *df* = 14). For EC trials there is a significant early to late increase when participants are primed clever (*p* = 0.01, *t* = 2.4, *df* = 14), but not when they are primed stupid (*p* = 0.22, *t* = 0.80, *df* = 14). For CC trials there is a significant early to late increase when participants are primed stupid (*p* = 0.03, *t* = 2.03, *df* = 14), but not when they are primed clever (*p* = 0.39, *t* = 0.30, *df* = 14). We refer to this as an early to late double dissociation. RTs for EC and CC trials are shown in Figure 5.

It is only when looking at performance over time that we can disentangle the differences between the two mental states. Looking at the overall RTs for CC trials there was no significant difference between stupid and clever (871 ms versus 905, *p* < 0.14, *t* = 1.09, *df* = 29). For EC trials there was a trend for stupid being shorter than clever (773 ms versus 842, *p* < 0.07, *t* = 1.51, *df* = 29).

#### 3.1.4 Correlation between psychometric scores and behavior

We did not find any significant correlation between psychometric scores and behavior. Difference in error rate between late and early sessions after “stupid”-priming (Rosenberg: *r* = −0.26, *p* = 0.36; STAI: *r* = 0.11, *p* = 0.71), the mean error-rate after “clever”-priming (Rosenberg: *r* = −0.32, *p* = 0.25; STAI: *r* = −0.42, *p* = 0.17), the difference in RT between late and early sessions after “stupid”-priming (Rosenberg: *r* = −0.11, *p* = 0.71; STAI: *r* = 0.24, *p* = 0.45).

### 3.2 Modeling results

We first present a number of qualitative features of the model, and then make inferences about model parameters from data fitting.

#### 3.2.1 Inferring faulty accumulation

**Figure 6:**
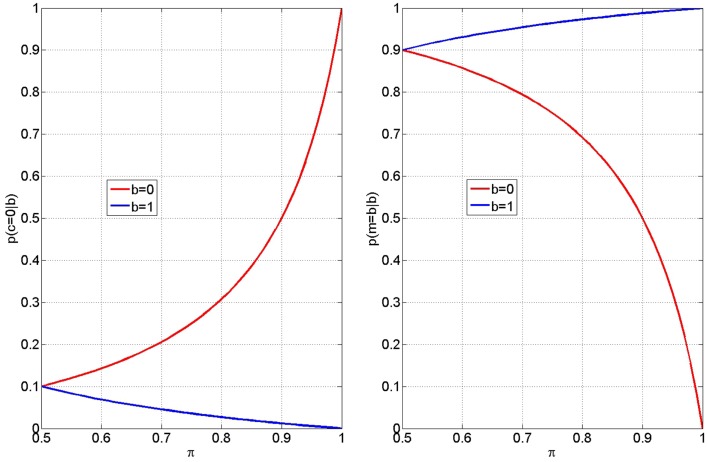
**Inferring faulty accumulation (left) participants are more likely to attribute errors (*b* = 0) to incorrect accumulation (*c* = 0) for larger values of π (red curve)**. Conversely, participants are more likely to attribute correct responses (*b* = 1) to incorrect accumulation for smaller values of π (blue curve). These curves were computed from equation (3) (red) and equation (4) (blue) with a value of β*_t_* = 0.9. The blue and red curves take on values 0 and 1 at π = 1 (perfect rule memory). The value 1 − β_*t*_ determines the intercept at π = 0.5 and thus controls the gradient of each of the above effects. Me-focus (Right) The extent of a subjects “me-focus” is quantified by the probability *p*(*m* = *b*—*b*) shown here subsequent to error outcomes, *b* = 0 (red curve) and correct outcomes, *b* = 1 (blue curve).

Equations (3 and 4) give the probability of inferring that the process of integrating the visual cue with memory for the rule (the response accumulation process) was faulty, given an error or a correct response on that trial. Figure 6 (Left) shows how these two probabilities, *p*(*c* = 0—*b* = 0) and *p*(*c* = 0—*b* = 1), vary with memory integrity, π. From the red curve it can be seen that participants will be more likely to attribute an error (*b* = 0) to faulty response accumulation (*c* = 0) as π increases. Similarly, from the blue curve, we see they will be more likely to attribute a correct response (*b* = 1) to faulty response accumulation as π decreases. If we assume that priming influences π, and further that “stupid” priming reduces π and “clever” priming increases it, then the above mechanism will cause the double dissociation observed in the early-late RT data. The negative slope of the blue curve then provides a simple explanation of the negative correlation between RT after corrects and correct rate (see Section 3.1.2).

#### 3.2.2 Me-focus

The extent of me-focus is quantified by the probability *p*(*m* = *b*—*b*), given in equations (7 and 8), and shown in Figure 6 (Right), subsequent to error outcomes (red curve) and correct outcomes (blue curve). Increasing π increases me-focus after correct and decreases me-focus after error. Thus, when primed clever, the attribution of an outcome to the self is increased after correct and decreased after an error.

#### 3.2.3 Fitting group data

**Figure 7:**
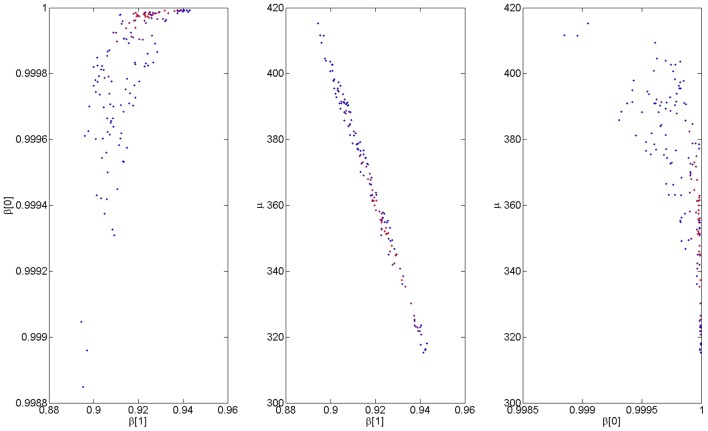
**Estimated model parameters from fitting group data**. The figures show samples from the bivariate posterior densities *p*(β[1], β[0]—*b*, *y*) (left), *p*(μ, β[1]—*b*, *y*) (middle), and *p*(μ, β[0]—*b*, *y*) (right). The color coding indicates model accuracy, with red indicating a better fit.

We now focus again on a main empirical finding of the paper; that for the stupid prime, the RTs after correct responses are negatively correlated with correct response rate (see Section 3.1.2). We fitted the computational model to this data by regressing RTs *y* onto correct rates, *r*, as described in Section 2.6. A Metropolis-Hastings procedure was used to obtain 10,000 samples from the posterior density *p*(β[1], β[0], μ—*y*, *r*). Figure 7 shows 1000 samples from this posterior; the ones that produce the best 10% of model fits. The middle plot shows a negative posterior correlation between μ and β[1]; i.e., the same model fit can be achieved by increasing μ and decreasing β[1]. Nevertheless, we can be confident that, e.g., 0.9 < β[1] < 0.94 and 310 < μ < 420. Importantly, we can be highly confident that β[0] > β[1] indicating that inferred faulty response accumulation does indeed cause an increase in the decision threshold for the next trial. In what follows we use the high probability parameter values β[1] = 0.92, β[0] = 0.9998, and μ = 360.

#### 3.2.4 Confirmation bias

If participants act as ideal Bayesian observers and update their beliefs about π then one will observe a confirmation bias effect. For example, if priming acts to induce a prior distribution over π and this prior places more probability mass on smaller values of π than does the likelihood, then posterior values will be lower than the maximum likelihood value. If this Bayesian updating mechanism operates, e.g., between sessions then the performance in the second session will be worse than in the first. In other words, if you think you’re going to do badly then you will.

We provide a numerical example of confirmation bias based on data from a single participant (participant 11). We used outcome data *b* from the first session for when this participant was primed stupid. We used the β values obtained from the group parameter estimation (previous section). The likelihood *p*(*b*—π) = *p*(*b*—π,β) can be computed as described in Section 2.5.

**Figure 8:**
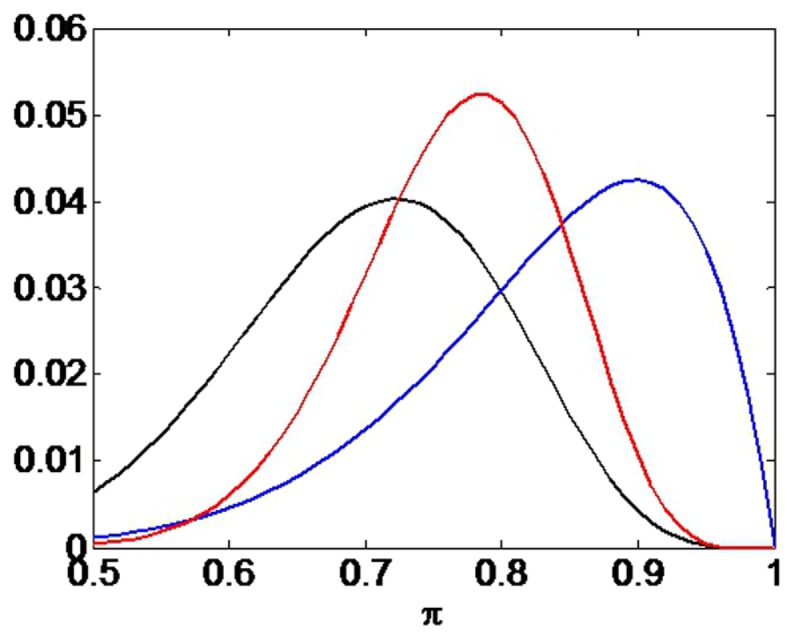
**Confirmation bias**. The black curve shows the prior *p*(π), the blue curve the likelihood *p*(*b*—π), and the red curve the posterior *p*(π—*b*). The curves have been scaled (on the *y*-axis) for ease of comparison. If the prior places more probability mass on smaller values of π than does the likelihood (e.g., through “stupid” priming) then the posterior will take on lower values than the maximum likelihood value.

The temporal scale of hypothesized Bayesian updating in the brain is unknown. It could happen discretely after each session or may be a slow process that is continually in operation. In our numerical example we compute the likelihood based on the first 10 outcomes only (9 of which were correct). This is for numerical convenience only as this small number of outcomes allows us to use the exact likelihood rather than an approximation to it (see Section 2.5). This likelihood is plotted as the blue curve in Figure 8.

We then hypothesize that the stupid prime takes the form of the black curve in Figure 8. This was implemented using a beta density (Bernardo and Smith, 1993). The posterior density *p*(π—*b*) is then computed using Bayes rule and is shown as the red curve, which clearly exhibits a confirmation bias. The particular form of the prior density here, e.g., beta density, is unimportant for our argument. As longs as the prior places more probability mass on smaller values of π than does the likelihood, a confirmation bias will ensue.

#### 3.2.5 Fitting individual subject data

**Figure 9:**
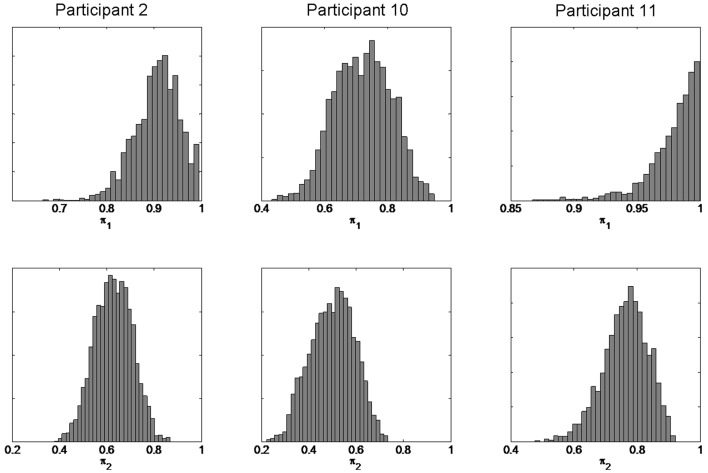
**Estimated model parameters from individual subject data**. The figure shows the posterior densities *p*(π_1_—*b*, *y*) (top row for session 1) and *p*(π_2_—*b*, *y*) (bottom row for session 3) for three participants.

We first present individual subject results for the three participants showing the largest confirmation bias effect (participants 2, 10, and 11). Figure 9 shows the posterior distributions for the π_1_ and π_2_ parameters. We can be confident that π_2_ < π_1_ for each participant. This shows that the confirmation bias effect is consistent with a reduction in π, i.e., is consistent with a priming effect mediated by a change in π.

We then used a two-way paired *t*-test to test whether memory integrity varied over sessions for the group of 15 subjects. This analysis was based on the posterior mean estimates of memory integrity. For the stupid prime data there was a significant difference between sessions (mean π_1_ = 0.88, mean π_2_ = 0.80, *p* = 0.02, *t* = 2.63, *df* = 14). For the clever prime data there was no significant difference between sessions (mean π_1_ = 0.85, mean π_2_ = 0.88, *p* = 0.25, *t* = 1.2, *df* = 14). This confirms our hypothesis that priming affects memory integrity: stupid priming significantly reduces π whereas clever priming does not significantly affect it.

## 4 Discussion

In this paper we provide novel behavioral findings of how attention to the cognitive task can be changed depending on which self-concept is currently active in mind. We observed a double dissociation between outcome (errors/corrects) and prime (clever/stupid). We augmented this finding by providing empirical evidence that Bayesian principles can be applied to self-regulatory processes such as “feeling stupid” and “feeling clever.” This strengthens the theory that, in the healthy individual, inner standards of ability beliefs are clearly defined structures (Kelly, 1955; Markus, 1977). Our findings are important because they demonstrate that the way self-concepts regulate behavior is based on the same general principles that guide decision-making processes on many levels (Doya et al., 2007).

Our model is simple in that it make use of two processes. One process refers to remembering which rule is active. This is a working memory process and as such it is a top-down process. The other process refers to the motor system integrating information from working memory with information from the visual system about which cue is present. The basic model then derives from the logic of the experimental task, whereby the task can be implemented correctly if both processes are either correct or if both are incorrect. As these processes are correctly implemented with some level of probability, Bayesian inference can be used to quantify the effects of changing these probabilities. Additionally, the model also incorporates an adaptive decision threshold based on Bayes rule, whereby decision thresholds are increased if integration was inferred to be faulty. We refer to this as Bayesian Adaptive Thresholding (BAT).

This paper hypothesized that priming affects the memory process. We were able to test this hypothesis by fitting our computational model to subjects’ behavioral data. Our results showed that stupid priming significantly reduces memory integrity (π) whereas clever priming does not significantly affect it. The Bayesian Adaptive Thresholding scheme, combined with the effect of priming on memory, then explains a main experimental finding of the paper; the early-to-late double dissociation in reaction times.

When participants are primed “clever” and make an error they are more likely to attribute the mistake to a fault in the motor-integration process rather than the working memory process, and will increase their decision threshold on the next trial so as to increase the likelihood of correct evidence accumulation. This naturally leads to a longer RT after an error response. Conversely, following priming with associations to “stupid,” the participants believe they have worse memory, and are therefore more likely to attribute an error to a fault in the working memory process. As a consequence, they will not increase their decision threshold on the next trial and thus not delay RT. The uncertainty in top-down processing seen for “stupid” makes them more likely than those primed “clever” to attribute correct responses to a “fluke,” in which both the working memory and the evidence accumulation processes are considered faulty. They tend to increase their decision threshold, and produce a longer RT on the next trial.

The lack of confidence in the participants’ memories after “stupid”-priming also explains why if you think you are going to do badly then you will (the confirmation bias); they should slow down after making an error but do not, and so continue to make errors. In other words, prior beliefs about performance are combined with estimates of actual performance (the “likelihood”) to set future levels of memory performance that are consistent with both. Since the priming is implemented via this prior, if the participants act as ideal Bayesian observers, their future performance will be determined by how the prior is affected by the prime. When participants have been primed “stupid” they place more probability mass on smaller values of memory integrity. Since this prior here places lower values on memory performance than does the likelihood, the performance deteriorates over the course of the experiment. This is analogous to leading psychological theories of self-regulation which stipulate that for consistency and predictability of self the discrepancy between self-ability beliefs and behavior is reduced (Bandura, 1982; Carver and Scheier, 1998). These theories are based on numerous behavioral findings, one example being an experiment where women who were told that females perform badly on math tasks then went on to perform worse than they would otherwise do (Spencer et al., 1999). We did not observe a change in performance level after “clever”-priming and suggest that this is because performance was commensurate with their beliefs; they did well and expected to do so.

We find further that when participants are primed “clever” they readily switch between attributing the cause of outcome to either of the processes. When the same participants are primed with associations of being “stupid” inferring the cause of outcome becomes less distinct. A complementary view on the inferential process subsequent to an outcome is the extent to which a participant identifies themselves with the outcome of a trial. Since memory integrity is in concordance with the influence of the prime it can be assigned “me-focus.” The integration process on the other hand, is a system which is not directly affected by priming, and can therefore be interpreted as “task-focused.” When associations to “clever” are active, in situations of making errors, the participants will readily reduce their me-focus and place emphasis on task processes; a mechanism that may reflect the discrepancy between their expectation and their actual performance. When making errors following “stupid”-priming the participants are more likely to attribute errors to a faulty memory process, i.e., me-focus, as well as to think of correct responses as flukes. This finding is supported by previous studies showing that depressed individuals (Greenberg and Pyszczynski, 1986) and low self-esteem individuals (DiPaula and Campbell, 2002) are more likely to persist in higher levels of self-focus after failure over time. Our model assumes the existence of these two processes but we have not directly observed them. The causes underlying the behavioral differences elicited by our priming study can, more generally, be described in terms of state characteristics and more enduring characteristics. Here the enduring characteristics are defined as stable, relatively general characteristics of the self that are consistent across situations, whereas states are transient characteristics that can change from moment to moment. Our model allows us to operationalize these definitions such that the “state” corresponds to the inferred state of the memory process (correct or not) and integration process (correct or not). The state thus changes from trial to trial. Whereas, the more enduring characteristic corresponds to the subject’s memory integrity, which changes on a longer time scale (e.g., session to session).

This paper has focused on the effect of priming on a very specific behavior – performance in a rule-association task. However, we have reason to expect that our computational approach will itself generalize, or can easily be generalized, to other behaviors. The core ideas of our approach are that (i) task performance depends on two component processes: a memory process and an evidence accumulation process, and that multiple combinations of the component processes can produce a correct outcome (e.g., both correct or both incorrect) (ii) after a trial, Bayesian inference is used to infer whether evidence accumulation was correct, and the decision threshold for the next trial is set accordingly. This model could be directly applied for example, to 1-back working memory tasks (match current to previous item), or the AX continuous performance tasks (press left if X follows A – the “target” is AX, right otherwise; Braver and Cohen, 2000). For more complex tasks such as n-back working memory (n > 1) one may conceive of multiple memory processes (one for each of the n previous items) instead of a single memory process. Or for the “12AX” task (if the last numeral you saw was a 1, the target sequence is “AX,” if a 2 its “BY”; Frank et al., 2001) we may again need multiple memory processes (one for last letter, one for last numeral). Nevertheless, for all of these cases, it will be possible to use Bayes rule to infer whether the evidence accumulation process was correct and so derive an appropriate Bayesian Adaptive Thresholding scheme.

One weakness of our study is that we did not use a reaction time model derived from optimality principles, rather we simply assumed that RTs were normally distributed with a mean reaction time being proportional to the log-odds ratio of the decision threshold. This is known to be a correct assumption for 2AFC tasks, and the rule accumulation task (Bengtsson and Penny, 2013). Our decision to use this rather simple model was motivated by the fact that the focus of this paper is on between-trial rather than within-trial dynamics (i.e., investigation of BAT scheme and effect of priming). Nevertheless, it will be possible in future studies to replace the simple Gaussian reaction time model with ones derived from optimality principles. These are now available, for example, for the Eriksen Flanker task (Liu et al., 2009), the Stop-Go task (Shenoy and Yu, 2011), and we are currently working on the rule-association task (Bengtsson and Penny, 2013).

The switching dynamics that our model portrays after “clever”-priming resemble the pattern of transient brain activation of the aMPC observed in Bengtsson et al. (2011) where activation goes up for errors and down for correct responses when participants are primed “clever.” It is interesting to note that the priming impacts on the confidence in memory processes, which themselves are processed on the aMPC (Summerfield et al., 2009). Our model suggests that the enhanced activation seen in aMPC after “clever”-priming when participants make errors reflects a switch away from me-focus to task-focus. That this signal only occurs when there is a positive expectation on performance is in line with findings that this area is signaling errors when participants are more motivated to do well on a task (Bengtsson et al., 2009), and when the errors reinforce individuals’ optimism (Sharot et al., 2011). Taken together, it suggests that self-related activation of aMPC that occurs during errors reflects processes of discrepancies when it is relevant to sustain positive aspects of self.

We have no direct evidence in this study that the priming actually targets self-concepts. According to the misattribution theory, primes will target our self-concept if we put ourselves in focus. This was empirically tested in a study where participants after trait-priming were asked to focus either on themselves or on someone else. In the self-focus condition the participants behaved in line with the prime and explicitly rated themselves in line with the prime, while these effects were absent in the other-focus condition (DeMarree and Loersch, 2009). The double dissociation observed in the present study between primes and the participants’ reactions to their own outcome (errors/corrects) would be difficult to explain if the prime simply targeted, e.g., semantic representations or associations to others, and supports the notion that in our study the participants’ self-concept was affected. There are studies showing a relationship between participants’ explicit self-ratings and their implicit associations in that a conflict between the two results in reduced task accuracy (Dislich et al., 2012) or increased bias in judgments Bosson et al. (2003). We did not find significant correlations between the psychometric scores and our participants’ error-rate or RT. The lack of significant results could be because explicit general self-esteem and reported anxiety are aspects which do not impact on processes important for the rule-switching task. In fact, Dislich et al. (2012) reported an interaction between implicit self-associations and explicit measures concerning “intelligence” in particular, but not for self-esteem in general.

In this paper we have highlighted the impact psychological factors can have on decision-making systems. We find that a contributing factor to optimal cognitive control is the implicit associations that people make to themselves as being clever. Our model suggests that these top-down associations regulate the efficiency of attentional switching between one’s own abilities and the task, as well as the confidence in one’s own memory processes.

## 5 Conflict of Interest Statement

The authors declare that the research was conducted in the absence of any commercial or financial relationships that could be construed as a potential conflict of interest.
